# Modulations of stretch reflex by altering visuomotor contexts

**DOI:** 10.3389/fnhum.2024.1336629

**Published:** 2024-02-14

**Authors:** Sho Ito, Hiroaki Gomi

**Affiliations:** ^1^NTT Communication Science Laboratories, Nippon Telegraph and Telephone Corporation, Atsugi, Japan; ^2^School of Engineering, Tokyo Institute of Technology, Yokohama, Japan

**Keywords:** stretch reflex, visually guided reaching, visuomotor transformation, state estimation, feedback control

## Abstract

Various functional modulations of the stretch reflex help to stabilize actions, but the computational mechanism behind its context-dependent tuning remains unclear. While many studies have demonstrated that motor contexts associated with the task goal cause functional modulation of the stretch reflex of upper limbs, it is not well understood how visual contexts independent of the task requirements affect the stretch reflex. To explore this issue, we conducted two experiments testing 20 healthy human participants (age range 20–45, average 31.3 ± 9.0), in which visual contexts were manipulated in a visually guided reaching task. During wrist flexion movements toward a visual target, a mechanical load was applied to the wrist joint to evoke stretch reflex of wrist flexor muscle (flexor carpi radialis). The first experiment (*n* = 10) examined the effect of altering the visuomotor transformation on the stretch reflex that was evaluated with surface electromyogram. We found that the amplitude of the stretch reflex decreased (*p* = 0.024) when a rotational transformation of 90° was introduced between the hand movement and the visual cursor, whereas the amplitude did not significantly change (*p* = 0.26) when the rotational transformation was accompanied by a head rotation so that the configuration of visual feedback was maintained in visual coordinates. The results suggest that the stretch reflex was regulated depending on whether the visuomotor mapping had already been acquired or not. In the second experiment (*n* = 10), we examined how uncertainty in the visual target or hand cursor affects the stretch reflex by removing these visual stimuli. We found that the reflex amplitude was reduced by the disappearance of the hand cursor (*p* = 0.039), but was not affected by removal of the visual target (*p* = 0.27), suggesting that the visual state of the body and target contribute differently to the reflex tuning. These findings support the idea that visual updating of the body state is crucial for regulation of quick motor control driven by proprioceptive signals.

## 1 Introduction

Humans can precisely execute various actions even in noisy and dynamically changing environments. The brain achieves such stable actions through a quick error correction mechanism using sensory information, namely, feedback control (Evarts and Tanji, 1976; Marsden et al., 1976; Goodale et al., 1986; Scott et al., 2015). Of the multiple feedback loops in our sensorimotor system, the stretch reflex plays a key role since it can correct sudden postural changes in response to proprioceptive input detected by a muscle spindle (Marsden et al., 1976; Pruszynski and Scott, 2012). Earlier studies have shown context-dependent modulation of the long-latency component of the stretch reflex recorded from wrist (Forgaard et al., 2015; Weiler et al., 2015) and arm muscles (Hammond et al., 1956; Evarts and Tanji, 1976; Kimura et al., 2006; Kurtzer et al., 2008; Shemmell et al., 2009; Pruszynski et al., 2011; Weiler et al., 2015). These studies suggested that the amplitude of the stretch reflex can be functionally regulated depending on various factors, including the task goal (Hammond et al., 1956; Evarts and Tanji, 1976; Kimura et al., 2006; Pruszynski et al., 2011; Forgaard et al., 2015; Weiler et al., 2015), stability of the environment (Shemmell et al., 2009), and body posture (Kurtzer et al., 2008). Such a flexible reflex modulation is enabled by tuning the reflex gain through continuous monitoring of the body state and the external world. However, the brain mechanism underlying this state monitoring for reflex modulation is not yet fully understood (Cluff et al., 2015).

Although the stretch reflex is primarily driven by proprioceptive input, recent studies have explored the possibility that the response of upper-limb muscles is affected by visual information representing the body state (Crevecoeur et al., 2016; Oostwoud Wijdenes and Medendorp, 2017; Kasuga et al., 2022) as well as other physical factors. With respect to voluntary movements, the integration of multiple sensory information minimizes uncertainty in state estimation, which contributes to the perception of body state and accurate motor control (Ernst and Banks, 2002; van Beers et al., 2002; Ronsse et al., 2009). Considering this, it is possible that the brain also uses the visual cue regarding self-body state to reduce state uncertainty for effective regulation of the stretch reflex. Actually, we have demonstrated that the stretch reflex of wrist muscles is regulated under several conditions when visual feedback of the hand position is distorted or not fully available (Ito and Gomi, 2020). Specifically, our findings indicate that the stretch reflex is attenuated when a visual feedback cursor is presented in spatial coordinates different from the actual hand movement, or when the visual cursor is partially or completely removed. While these results suggest significant involvement of visual feedback in the modulation of the stretch reflex, a detailed account remains unclear. To clarify the critical factors in the vision-dependent modulation of the stretch reflex, the present study addressed the following two issues.

First, our previous study suggested that manipulating the spatial configuration between hand movements and the visual cursor impacts the hand muscle stretch reflex. Specifically, the amplitude of the stretch reflex decreased when the hand movement was transformed into the visual cursor by rotation or mirror-reversal. This finding suggests that a large visuomotor discrepancy causes the reduction of the stretch reflex gain, implying that the brain tends to suppress the large motor correction in such a situation. However, it is currently unknown what factor of visuomotor transformations is responsible for regulating the stretch reflex. It is common for humans to perform movement tasks with visual feedback that is displayed in spatial coordinates different from those of the body’s movement. If the movement task is performed in a familiar or sufficiently learned spatial configuration, such as operating a mouse to control a cursor on a computer monitor, the task can be completed without difficulty owing to the acquired visuomotor mapping between the body states and the visual cursor (Messier and Kalaska, 1997; Gorbet et al., 2004). Meanwhile, if bodily movements are transformed into the movements of the visual cursor by a novel or unfamiliar mapping, the motor control is imprecise and unstable due to an insufficient estimate of the body state by using visual information (Abeele and Bock, 2001; Bernier et al., 2009; Ito and Gomi, 2020). Assuming that uncertainty in the body state impacts the stretch reflex, we predicted that the amplitude of the stretch reflex is regulated depending on whether a visuomotor mapping has been acquired or not. To test this hypothesis, we first investigated the following two types of visuomotor transformation and their effect on the stretch reflex: (1) a visual cursor displayed with rotation from the actual body movement, but appearing unchanged in the visual coordinates due to simultaneous head rotation, and (2) visual feedback rotation occurring on the visual coordinates. Considering that visual coordinates have essential roles in motor planning and estimation of the online state for visually guided reaching (Lacquaniti and Caminiti, 1998; Buneo et al., 2002; Buneo and Andersen, 2006), we assumed that whether feedback representation is maintained in visual coordinates affects how the brain updates the visuomotor mapping.

Second, while our previous study suggested that the gain of the stretch reflex is modulated depending on the uncertainty in the hand state, it has remained unclear whether uncertainty in the visual target location also affects the stretch reflex. Though earlier studies have demonstrated a task-dependent regulation of the arm muscle stretch reflex in response to the size (Yang et al., 2011) or position (Mutha et al., 2008) of the visual goal, the effect of visual target uncertainty on the stretch reflex has not been investigated. Furthermore, previous studies have shown that uncertainty in the visual target induced by visual noise (Izawa and Shadmehr, 2008) or dynamic location updates (Dimitriou et al., 2013; Abekawa and Gomi, 2015) affects the online corrective visuomotor response. However, it remains unclear whether uncertainty in the visual target affects the gain of the quick feedback control evoked by sensory inputs in different modalities. To clarify this point, we examined whether the size of the stretch reflex is impacted by manipulating the visibility of the target in addition to online visual feedback of the hand position.

Through the two experiments, we tested the hypothesis that the amplitude of the stretch reflex is regulated considering the reliability of the body state obtained by integrating multisensory information, including visual cues. To this end, we manipulated visual contexts that significantly influence body state estimates (namely, visuomotor mapping and the appearance of the online visual feedback), while keeping other factors (e.g., motor state and task goal) unchanged. The results would provide clues to reveal the brain process that allows flexible and functional modulation of feedback control depending on the online representation of the body state.

## 2 Materials and methods

In total, 20 healthy volunteers (8 males, 12 females; age range 20–45, average 31.3 ± 9.0) participated in two experiments. All of them were right-handed. All gave written informed consent to participate in the experiments. Ten of them participated in Experiment 1, and the other ten participated in Experiment 2. The number of participants (sample size) was determined by referring to previous studies of stretch reflex modulation (Kimura et al., 2006; Kurtzer et al., 2008). The experimental protocol was approved by a local ethics committee. Each experimental session took about 3 h including preparation.

**FIGURE 1:**
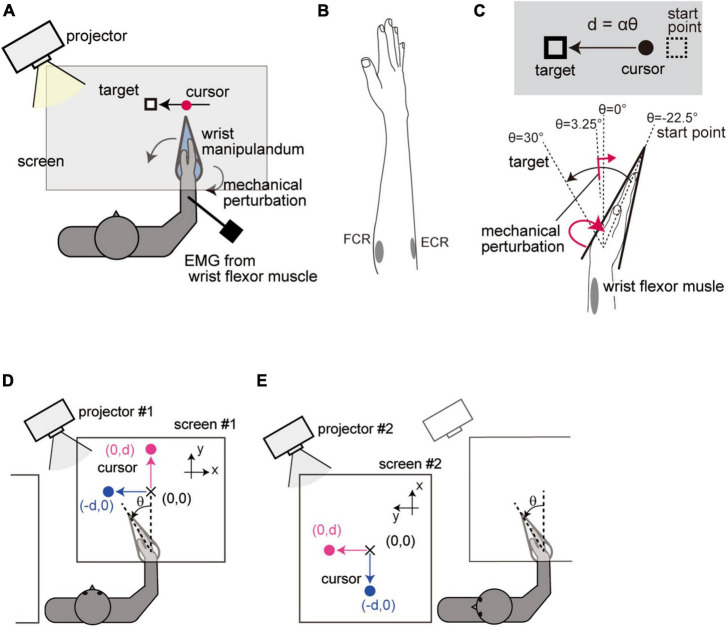
Setup of Experiment 1. **(A)** Apparatus. **(B)** Placement of electrodes. **(C)** Spatial configuration of the movement task. Visual feedback of the hand movement was displayed on the screen as a linear displacement of the cursor. The cursor position *d* was calculated from the wrist flexion angle θ from a straight hand posture according to the equation *d* = αθ, where α(0.44 cm/deg) is the visual feedback gain. **(D)** Standard configuration. Wrist movement was displayed as a horizontal displacement of the cursor (blue) in the Horizontal-VF trials and as a vertical displacement in the Vertical-VF trials (magenta). **(E)** Head-rotated configuration. Participants performed the task while looking leftward of the body by rotating the neck. The whole visual workspace was displayed with a 90° rotation around the participants’ head axis so that the visual feedback was kept unchanged in visual coordinates from the Standard configuration.

Participants were seated in a chair facing a horizontally set screen (Figure 1A). Their right hand was tightly fixed with a strap to a custom-made wrist manipulandum (maximum torque of 7.0 Nm). The manipulandum allowed one degree-of-freedom rotation in a horizontal plane, limiting the hand movement to only flexion and extension of the wrist joint. Their right forearms were fastened by a soft belt to an armrest. The participants’ hand was occluded by a screen, and instead, feedback of the hand movement was provided by a visual cursor displayed on the screen via a projector (K335, Aser Inc., New Taipei City, Taiwan) at a refresh rate of 60 Hz. The position of the visual cursor was calculated from the wrist flexion angle, which was recorded at 500 Hz with a rotary encoder (resolution of 0.0055°) attached to the manipulandum. Electromyography (EMG) data were measured (Figure 1B) from the right wrist flexor (FCR: flexor carpi radialis) and extensor muscle (ECR: extensor carpi radialis) by using surface electrodes (Ag-AgCl disposable electrode, GE Healthcare Japan, Tokyo, Japan). The electrode placements were determined by palpation of the muscle belly while pushing against a wall with the palm (FCR) and back of the hand (ECR). The signal was amplified (MME-3116, Nihon Kohden, Tokyo, Japan), filtered (0.53–1000 Hz), and then sampled at 2000 Hz. The hand position, EMG signals, and the control signal of the manipulandum were recorded synchronously with a digital signal processor system operating at a control frequency of 2000 Hz (iBIS DPS7101A, MTT Co., Tokyo, Japan) controlled by custom software. Visual and auditory stimuli were controlled by MATLAB (Mathworks Inc., Natick, MA, USA) and Cogent graphics toolbox (developed by John Romaya at the LON at the Wellcome Department of Imaging Neuroscience).

### 2.1 Experiment 1

The purpose of Experiment 1 was to investigate how the stretch reflex is impacted by two different types of rotational visuomotor transformation. First, participants were instructed to perform the reaching task under visual feedback rotation while rotating the head. We compared the stretch reflex under the two configurations (Standard vs. Head-rotated configuration) to test whether it is regulated by changing the mapping between the arm movement and visual feedback while maintaining the feedback configuration in visual coordinates. Second, the stretch reflex was also compared between conditions in which the visual feedback was rotated or not (Horizontal-VF vs. Vertical-VF) to examine the effect of an additional rotation of the visual feedback relative to visual coordinate frame.

The visual feedback of the hand position was provided as a cursor that moved linearly. The wrist flexion angle θ was transformed into a linear cursor displacement *d* according to the equation *d* = αθ (Figure 1C). Here, the constant α was set to 0.44 [cm/deg]. Participants were required to reach the target location (θ = + 30°) guided by the visual cursor (Figure 1C). The starting point of the movement (equivalent to θ = −22.5°) was displayed as a small rectangle before each trial. When the participant moved the visual cursor onto the starting point, a visual target (same rectangle as the starting mark) immediately appeared, and three beeps (ITI = 750 ms) were given as notification of the timing of the movements. Participants were instructed to start their reach at the second beep and complete it at the third beep.

**FIGURE 2:**
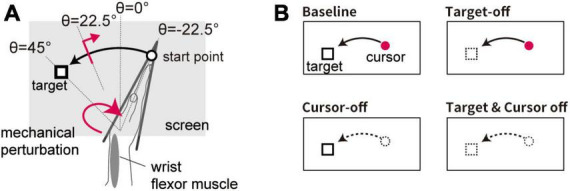
Setup of Experiment 2. **(A)** Spatial configuration of visual stimuli for the movement task. Visual feedback of the hand movement was given as a cursor displayed at the fingertip position. **(B)** Schematic diagram of visual feedback condition. Four types of trial emerge from manipulating the appearance of the visual cursor and target (cursor on/off × target on/off).

As one type of manipulation, we altered the spatial relationship between the arm movement and visual feedback while keeping the configuration of the feedback constant in visual coordinates. To achieve that, we simultaneously introduced a rotation of visual feedback and corresponding head rotation. Participants performed the reaching task in (1) a condition where visual feedback was displayed in front of their body (Standard configuration, Figure 1D) and (2) a condition where visual feedback was displayed on the left side of the participants’ body while they rotated their head 90° to the left (Head-rotated configuration, Figure 1E). In the Head-rotated configuration, the whole visual information was rotated by 90° around the center of the participants’ head so that the visual workspace remained unchanged in the visual coordinates across the configurations. These conditions were altered by switching between two sets of a projector and a screen.

As the other type of manipulation, we tested the effect of a visual feedback rotation with respect to visual coordinates. To this end, participants were further asked to perform two different types of trials regarding the direction of cursor movement in each postural configuration. In one condition, the hand position was provided as a transversal displacement of the cursor from the participants’ point of view (Horizontal-VF trial, blue in Figures 1D, E), while in the other condition, the hand position was represented as a longitudinal displacement of the cursor (Vertical-VF trial, magenta in Figures 1D, E).

The four experimental conditions (2 postural configurations × 2 cursor directions) were tested as separated experimental blocks consisted of 24 trials. In 50% of the trials, the visual target remained at the initial location throughout the trial (Test trial). The stretch reflex was evaluated in half of the Test trials (see section “2.3 Reflex measurement”). In the other 50% of the trials, to prevent the participants from reaching without visual information, the target was abruptly shifted forward or backward with equal probability (equivalent to an angle change of ± 22.5°) when the hand passed a constant location (θ = + 3.25°) and participants had to reach the shifted target location (Catch trial). Indeed, the results revealed that the movement endpoints in the Catch trial were suitably shifted toward the jumped target locations (+ 22.45 ± 0.027° from the original target for a forward jump and −22.06 ± 0.027° for a backward jump), which indicated that the participants used online visual information to execute the reaching movements. The participants sequentially performed 4 experimental blocks (each containing 12 Test trials and 12 Catch trials, in random order) under all four experimental conditions (order was randomized) and repeated this four times (384 trials in total) with a short break in between.

### 2.2 Experiment 2

The aim of Experiment 2 was to test how uncertainty in the state of the visual cursor and target affects the modulation of the stretch reflex. To this end, the stretch reflex was evaluated under four different types of trials in which the cursor and/or target were removed on a trial-by-trial basis, thus manipulating uncertainty about the cursor and target locations.

In this experiment, the visual cursor moved along an arc with a radius of 15 cm depending on the flexion angle of the wrist (Figure 2A). This setting matched the displayed position of the visual cursor with the participants’ actual right fingertips. Participants were requested to flex their hand from starting point (θ = −22.5°) to target by using the visual feedback. Timing information was given by beep sounds in the same manner as in Experiment 1.

In 50% of the trials (Test trial), the visual target was displayed at a constant location (θ = + 45°). In half of the Test trials, the stretch reflex was evoked (see section “2.3 Reflex measurement”). In the remaining trials (Catch trial), the visual target appeared at a random location (selected from a uniform distribution: θ ϵ [+57 ° + 33]°) to prevent participants from making movements toward a memorized location without visual information.

In some of the trials, the visual cursor was eliminated after onset of movement (when the hand moved more than 1° from the starting point) in order to examine the effect of uncertainty in the self-state on the stretch reflex (Cursor-off trial). In addition, the effect of uncertainty in the target location was tested by presenting the visual target for a limited duration (for 100 ms) at the trial onset and subsequently removing it until completion of the movement (Target-off trial). The effects of these manipulations were tested in a 2 × 2 factorial design (cursor on/off × target on/off), namely using four trial types (Figure 2B). In all types of trial, the visual cursor and target were briefly displayed (∼170 ms) at the terminal position to provide feedback after the hand movement stopped. An experimental block consisted of 96 trials containing all four trial types (24 trials each), each including 12 Test trials and 12 Catch trials. The order of the conditions was randomized within each experimental block. Each participant completed the experimental blocks four times (i.e., 384 trials in total) with short breaks in between.

### 2.3 Reflex measurement

In both experiments, to evoke stretch reflex, a mechanical perturbation (half sine-wave torque pattern with a 50 ms duration and 2.0 Nm peak amplitude) was applied through the manipulandum in the middle of the wrist flexion movement. This perturbation was applied in the direction of wrist extension, which caused a rapid stretch of the wrist flexor muscle and evoked the stretch reflex. The perturbation was initiated when the hand passed predefined trigger positions (θ = + 3.25° in Experiment 1, the midpoint of the range of motion, and θ = + 22.5° in Experiment 2, the two thirds point of the range of motion). To keep the participants from making any anticipatory responses, the mechanical perturbation was given in randomly selected 50% of the Test trials. No perturbation was applied in the remaining Test trials or in the Catch trials. For each visual condition, data of all mechanically perturbed trials (24 trials) were used to analyze the stretch reflex.

### 2.4 Data analysis

The movement endpoint was defined as the position at which the velocity of the hand fell below a threshold (5% of the peak velocity in each trial) and remained there for the succeeding 200 ms. In addition to calculating the average value of the movement endpoints, the standard deviation was evaluated as an index of movement precision.

The digitized EMG signal was rectified after a high-pass filtering (using a zero-phase lag, fourth-order Butterworth filter with a 50-Hz cutoff frequency) to remove motion artifacts. The rectified EMG was aligned to the onset timing of the mechanical perturbation to calculate the intertrial average. To evaluate the amplitude of the stretch reflex, mean activity was calculated in constant time windows from the onset of the perturbation (short-latency response, from 30 to 50 ms; long-latency response, from 50 to 100 ms), referring the previous study (Lee and Tatton, 1982). The activity just before the perturbation (mean value calculated in a time window between −50 and 0 ms from the perturbation onset) was also evaluated as an index of background activity (Cluff and Scott, 2013). This pre-perturbation activity was subtracted from these reflex components to remove the effect of fluctuations in background muscle activity. For between-subject analysis, the amplitude of the muscle activity was normalized using reference activity during isometric contraction against reference torque of 1 Nm, which was recorded before starting the experiment.

To test for statistical differences in the reflex amplitude and the behavioral data (mean and standard deviation of movement endpoints, movement velocity, and movement duration) among the visual conditions, the results of Experiment 1 were analyzed with a two-way repeated measures ANOVA (analysis of variance) with factors of head rotation (Standard or Head-rotated configuration) and visual feedback rotation (Horizontal- or Vertical-VF). Similarly, the results of Experiment 2 were analyzed with a two-way ANOVA with factors of removing the cursor (cursor-on/off) and the target (target-on/off). If a significant interaction effect was found, a Tukey’s HSD test was conducted as a *post hoc* analysis. Before performing the ANOVA, data normality was checked with Lilliefors test. If the normality was violated, Wilcoxon signed-rank test was used instead to compare the variables between conditions. For all ANOVAs, data sphericity was verified with Mauchly’s test.

To remove outliers, unperturbed trials were discarded from the analysis if the movement duration or endpoint position was more than three times the median absolute deviation away from the median for each condition. Similarly, perturbed trials were discarded by examining the amplitude of the long-latency stretch reflex using the same criterion. In total, 3.6% trials were excluded, and the most trials removed from an individual was 7.3%.

## 3 Results

### 3.1 Experiment 1

We designed this experiment to examine how two different classes of visuomotor transformation affect the stretch reflex. First, we tested the effect of changing the mapping between the arm movement and visual feedback while maintaining the configuration of feedback in visual coordinates. For this purpose, we asked the participants to perform the reaching task under visual feedback rotation while rotating the head (Standard vs. Head-rotated configuration). Second, we examined the impact of an additional rotation of visual feedback with respect to visual coordinate frame (Horizontal-VF vs. Vertical-VF).

**FIGURE 3:**
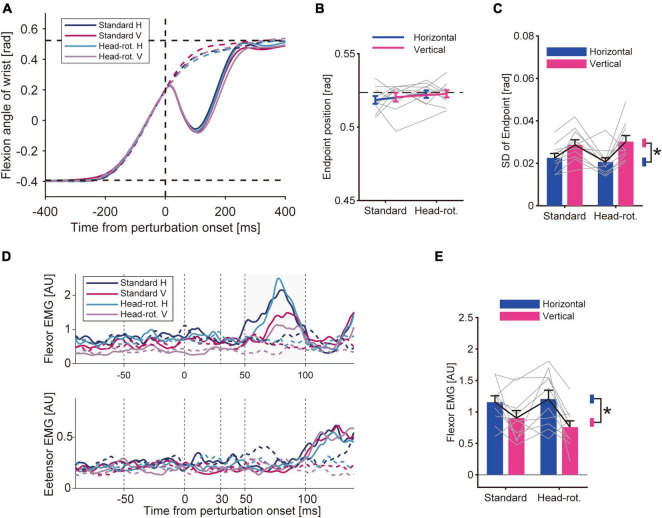
Results of Experiment 1. **(A)** Hand trajectory of representative participant. Data was averaged for each trial type after aligning it to the timing when the hand passed the constant location (θ = + 3.25°) for applying the mechanical perturbation. Solid curve: perturbed trials. Dotted curve: unperturbed trials. Two horizontal dotted lines mean the start and goal position. **(B)** Movement endpoints of unperturbed trials. Bars are group mean (*n* = 10), and gray lines are data of each participant. Error bars represent the standard error of the mean. The horizontal dotted line represents the displayed goal position. A two-way ANOVA did not show a significant effect of either head rotation (*p* = 0.096) or visual feedback rotation (*p* = 0.24). **(C)** Standard deviation of movement endpoint (group mean ± SE). A two-way ANOVA showed a significant effect of visual feedback rotation (*p* = 4.7 × 10^–3^). The effect of head rotation (*p* = 0.87) and the interaction of two factors (*p* = 0.28) were not significant. Asterisk represents the significant difference shown by the ANOVA. **(D)** EMG from wrist flexor (FCR) and extensor muscle (ECR) aligned to the onset of the mechanical perturbation. Data of a representative participant. Averaged data for each trial type are shown for the perturbed trials (solid curve) and unperturbed trials (dotted curve). **(E)** Amplitude of the long-latency stretch reflex (group mean ± SE). A two-way ANOVA showed a significant effect of visual feedback rotation (*p* = 0.024) while it did not show a significant effect of head rotation (*p* = 0.26) or interaction between these factors (*p* = 0.16).

Under the four different visual feedback conditions, the participants performed reaching movements by flexing their wrist. The movement trajectory of the unperturbed trials was similar across the visual conditions (Figure 3A, dotted curve). We did not find any statistical difference in the average movement endpoint (Figure 3B). The two-way ANOVA did not show any effect of either head rotation [*p* = 0.096, *F*_(9_, _1)_ = 3.46, partial η^2^ = 0.28] or visual feedback rotation [*p* = 0.24, *F*_(9_, _1)_ = 1.57, partial η^2^ = 0.018]. On the other hand, the movement precision was affected by the visual feedback condition (Figure 3C). The two-way ANOVA showed a significant effect of the visual feedback rotation on the standard deviation of the endpoint [*p* = 4.7 × 10^–3^, *F*_(9_, _1)_ = 13.94, partial η^2^ = 0.61], indicating that the endpoint variability was larger in the Vertical-VF trials than in the Horizontal-VF trials. Meanwhile, the ANOVA did not show significant effects of head rotation [*p* = 0.87, *F*_(9_, _1)_ = 0.030, partial η^2^ = 0.034] or interaction of these factors [*p* = 0.28, *F*_(9_, _1)_ = 1.33, partial η^2^ = 0.13] on the standard deviation of the endpoint. This suggests that the rotation of visual feedback in visual coordinates made the movement more variable, whereas a less clear effect was observed with the visual rotation accompanied by the head rotation that maintained the feedback representation in visual coordinates.

On randomly chosen trials, we applied a mechanical perturbation that rapidly extended the wrist. This perturbation caused a rapid stretch of the wrist flexor muscle and evoked the stretch reflex, which quickly compensated for the disturbance caused by the perturbation (Figure 3A, solid curve). Accordingly, the EMG trace showed a rapid increase in muscle activity within a short time range after the perturbation (Figure 3D). By comparing the size of the stretch reflex (see section “2 Materials and methods”) across conditions, we found a modulation of the amplitude of the long-latency response under the visual feedback condition (Figure 3E). The two-way ANOVA showed that the amplitude of the long-latency response was statistically smaller in the Vertical-VF trials than in the Horizontal-VF trials, as indicated by the significant effect of the visual feedback rotation on the amplitude [*p* = 0.024, *F*_(9_, _1)_ = 7.39, partial η^2^ = 0.45]. In contrast, the ANOVA did not show a significant effect of the head rotation on the amplitude of the long-latency response [*p* = 0.26, *F*_(9_, _1)_ = 1.44, partial η^2^ = 0.14], or any significant effect of the interaction between visual feedback rotation and head rotation [*p* = 0.16, *F*_(9_, _1)_ = 2.32, partial η^2^ = 0.20]. These results suggests that the amplitude of the long-latency stretch reflex was decreased by rotating visual feedback in visual coordinates while it was not affected by visuomotor transformation maintaining the feedback representation in visual coordinates. To examine the possibility that the observed stretch reflex modulation was attributed to a change in background muscle activity caused by the manipulation of visual context, we compared the EMG immediately before the onset of the mechanical perturbation (between −50 and 0 ms). This pre-perturbation activity of the flexor muscle did not statistically differ between the Vertical-VF and the Horizontal-VF trials [*p* = 0.12, *F*_(9_, _1)_ = 2.88, partial η^2^ = 0.24] while it was significantly smaller in the Head-rotated configuration than in the Standard configuration [*p* = 0.024, *F*_(9_, _1)_ = 7.27, partial η^2^ = 0.45]. These results do not account for the observed change in the long-latency stretch reflex. We also verified the pre-perturbation activity of the wrist extensor muscle and did not find significant difference between Standard and Head-rotated configuration (*p* = 0.30, *Z* = 1.05, *r* = 0.23) or Horizontal-VF and Vertical-VF trials (*p* = 0.91, *Z* = 0.11, *r* = 0.025), suggesting that the modulation of the stretch reflex is not explained by a change in co-contraction pattern. We did not find a significant effect from either type of visual manipulation on the amplitude of the short-latency stretch reflex (Standard vs. Head-rotated configuration, *p* = 0.88, *Z* = 0.15, *r* = 0.033; Horizontal-VF vs. Vertical-VF trials, *p* = 0.33, *Z* = 0.97, *r* = 0.22).

### 3.2 Experiment 2

**FIGURE 4:**
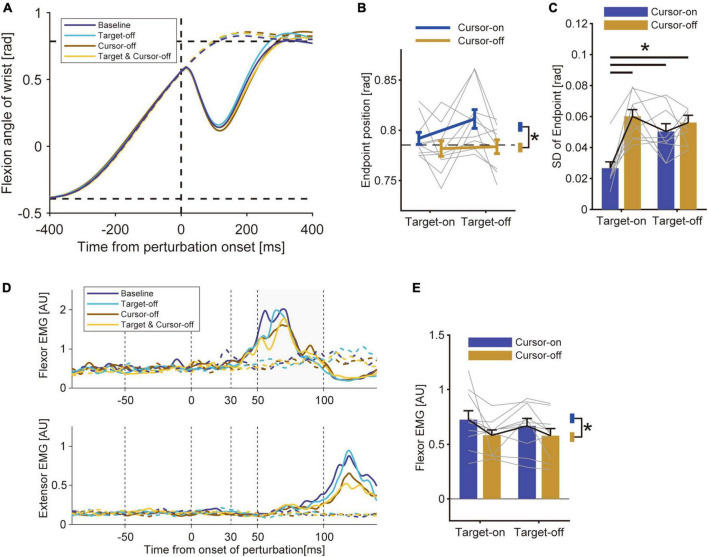
Results of Experiment 2. **(A)** Hand trajectory of a representative participant. Data were aligned to a timing at which the hand passed the constant location (θ = + 22.5°) where the mechanical perturbation was applied. Solid curve: perturbed trials; Dotted curve: unperturbed trials. **(B)** Movement endpoints of unperturbed trials (group mean ± SE). A two-way ANOVA showed a significant effect of cursor removal (*p* = 0.019). The effect of target removal (*p* = 0.18) and the interaction between these factors (*p* = 0.08) were not significant. Asterisk represents the significant difference shown by the ANOVA. **(C)** Standard deviation of movement endpoint (group mean ± SE). A two-way ANOVA showed a significant effect of cursor removal (*p* = 1.7 × 10^–3^), target removal (*p* = 0.039), and interaction (*p* = 3.6 × 10^–3^). Horizontal bars represent significant differences found by a *post-hoc* test (*p* < 0.05). **(D)** EMG from wrist flexor and extensor muscle (representative participant). Solid curve: perturbed trials; Dotted curve: unperturbed trials. **(E)** Amplitude of the long-latency stretch reflex (group mean ± SE). A two-way ANOVA showed a significant effect of cursor removal (*p* = 0.039). The effect of target removal (*p* = 0.27) and the interaction between these factors (*p* = 0.53) were not significant.

In Experiment 2, we tested how uncertainty about the visual cursor and target affected the modulation of the stretch reflex. To manipulate the uncertainty of the cursor and target locations, we presented or removed those visual cues in a trial-by-trial manner. The average reaching trajectory in the unperturbed trials did not largely differ across visual conditions (Figure 4A). A two-way ANOVA with factors of visual cursor and target did not show any significant effect of target removal on the average movement endpoint [Figure 4B, *p* = 0.18, *F*_(9_, _1)_ = 2.12, partial η^2^ = 0.19]. Though the removal of the visual cursor caused a slight but significant difference in the movement endpoint [*p* = 0.019, *F*_(9_, _1)_ = 8.11, partial η^2^ = 0.47], we did not find a significant effect on the movement duration (*p* = 0.53, *Z* = 0.63, *r* = 0.14) or peak velocity (*p* = 0.057, *Z* = 1.90, *r* = 0.43). We also evaluated the effect of the visual information on movement precision (Figure 4C). The two-way ANOVA revealed significant effects from both the visual cursor [*p* = 1.7 × 10^–3^, *F*_(9_, _1)_ = 19.30, partial η^2^ = 0.68] and the target [*p* = 0.39, *F*_(9_, _1)_ = 5.81, partial η^2^ = 0.39] as well as the interaction between them [*p* = 3.6 × 10^–3^, *F*_(9_, _1)_ = 15.24, partial η^2^ = 0.63] on the standard deviation of the movement endpoints. A *post hoc* analysis showed that endpoint variability was significantly larger in both Cursor-off trials and Target-off trials compared with baseline trials. The results indicate that increasing the uncertainty in both the visual cursor and the target degraded movement precision. We also found that movement variability was larger in the trials where both the cursor and target were removed, but the amount of increase was not different from in the trials where each factor was removed separately. Possibly, this lack of additive effect can be explained by the general ceiling effect.

By comparing the sizes of the stretch reflexes across conditions, we found a modulation of the amplitude depending on the visual feedback condition (Figure 4D). Specifically, the amplitude of the long-latency stretch reflex was smaller in the Cursor-off trials than in the Cursor-on trials, while it was comparable between the Target-off and Target-on trials (Figure 4E). Indeed, a two-way ANOVA revealed a significant effect of removing visual cursor on the size of the long-latency stretch reflex [*p* = 0.039, *F*_(9_, _1)_ = 5.82, partial η^2^ = 0.39], whereas it did not show any significant effect of target removal [*p* = 0.27, *F*_(9_, _1)_ = 1.37, partial η^2^ = 0.13] or interaction between these factors [*p* = 0.53, *F*_(9_, _1)_ = 0.42, partial η^2^ = 0.045]. This suggests that the increase in uncertainty about visual cue regarding the self-state suppressed the stretch reflex, but the ambiguity in the visual target location did not affect the amplitude. The pre-perturbation muscle activities were not statistically different by either type of visual manipulation for both the flexor (effect of cursor, *p* = 0.0072, *Z* = 2.69, *r* = 0.60; effect of target, *p* = 0.82, *Z* = 0.22, *r* = 0.050) and the extensor muscles (effect of cursor, *p* = 0.68, *Z* = 0.41, *r* = 0.092; effect of target, *p* = 0.94, *Z* = 0.075, *r* = 0.017). These results suggest that the modulation of the stretch reflex is not due to a change in background activity or co-contraction level. As in Experiment 1, we did not find any significant effect from either removing the visual cursor (*p* = 0.91, *Z* = 0.11, *r* = 0.025) or the target (*p* = 0.16, *Z* = 1.41, *r* = 0.32) on the amplitude of the short-latency stretch reflex.

## 4 Discussion

The present study investigated how visual contexts cause modulation of the hand muscle stretch reflex through two experiments. In Experiment 1, we examined the impact of a visuomotor transformation on the stretch reflex. We found that a rotational visuomotor transformation reduced the stretch reflex amplitude and movement precision, but the rotational transformation did not affect the stretch reflex or the motor performance when visual feedback was maintained in visual coordinates. In Experiment 2, we tried to determine whether visual uncertainty in the hand state and goal information affects the stretch reflex amplitude by eliminating the visual cursor and target during movements. The results showed that the cursor elimination led to a decrease in stretch reflex amplitude, whereas the target elimination did not affect the stretch reflex. These findings suggest that visual uncertainty in the self-body state is significantly related to suppression of the stretch reflex.

The previous study showed that introducing a rotational transformation on the hand cursor caused a reduction in the amplitude of the hand muscle stretch reflex (Ito and Gomi, 2020), but this finding has not had a conclusive interpretation. One possibility is that the reflex regulation occurs due to visual uncertainty in hand state estimates resulting from a novel visuomotor mapping; another potential account is that a spatial mismatch between visual and proprioceptive information may cause attenuation of the proprioceptive input, as suggested by earlier studies (Jones et al., 2001; Bernier et al., 2009). To elucidate this point, in Experiment 1, we applied two different types of visuomotor rotation in order to investigate their effect on the stretch reflex. Both types of manipulation induced a spatial mismatch at an angle of 90° between the movement of the hand and the visual cursor. However, one manipulation maintained the visual feedback representation in visual coordinates through an accompanying head rotation, whereas the other manipulation caused a rotation of visual feedback with respect to visual coordinates.

For the first type of manipulation (i.e., visual feedback rotation accompanied by a head rotation), we did not find any effect on movement precision compared with the condition without manipulation (Figure 3C). The results suggest that, in this condition, participants could successfully update their hand state estimates by using visual feedback and perform the movement task. This implies that when the movement direction of the visual cursor is maintained in visual coordinates, humans can keep the already learned visuomotor transformation by compensating for the change in the spatial mismatch between the body and visual feedback caused by head rotation. Previous studies have demonstrated that, in visually guided reaching movements, the target and hand positions are encoded in visual coordinates, and the representation is used for motor planning (Buneo et al., 2002; Bernier and Grafton, 2010). Further, this representation in visual coordinates is used for estimating the hand state by associating visual feedback with previous and ongoing motor commands (Buneo and Andersen, 2006). Given the critical role of visual coordinates in motor planning and state estimation, the results of the present study are reasonable. Notably, we did not find a significant change in the stretch reflex amplitude in this condition (Figure 3E). This suggests that as long as a visuomotor mapping is established in the brain, the stretch reflex is not affected even if visual feedback is provided at a location different from the actual hand position in the visual coordinates.

In contrast, the other type of visual manipulation (visual rotation with respect to visual coordinates) significantly increased movement variability (Figure 3C). As numerous motor learning studies have shown, visual rotation disrupts motor execution until the visuomotor mapping is updated by sufficient sensorimotor adaptation (Buch et al., 2003; Saijo and Gomi, 2010, 2012; Henriques and Cressman, 2012). Accordingly, we speculate that the visuomotor mapping was unlearned in this condition, and the participants could not accurately estimate the hand state from visual feedback. With this manipulation, we found a decrease in the amplitude of the long-latency stretch reflex (Figure 3E). The results were consistent with the hypothesis that the stretch reflex is modulated depending on the uncertainty in the hand-state estimate caused by novel or unfamiliar visuomotor transformations, rather than merely a spatial mismatch between visual feedback and proprioceptive signals.

Experiment 2 tried to determine whether introducing uncertainty to the visual cursor and target locations affects the stretch reflex. Previous studies have established the vital role of both hand cursor and target information in online control of visually guided movement (Soechting and Lacquaniti, 1983; Day and Lyon, 2000; Saunders and Knill, 2004; Franklin et al., 2016). Indeed, the present study showed that removing these visual stimuli significantly increased movement variability (Figure 4C), indicating that uncertainty in the positions of both the visual cursor and the target impaired movement performance. However, removal of one uncertainty had a different effect on the amplitude of the stretch reflex compared with removal of the other (Figure 4E). In particular, we found that hiding the visual cursor decreased the amplitude of the long-latency stretch reflex, which supports the account that the brain tunes reflex gain depending on the reliability of the body state estimated from vision and other sensory information (Ito and Gomi, 2020). In contrast, we found that removing the visual target did not affect the amplitude of the stretch reflex. Several studies have demonstrated a modulation of the upper-limbs long-latency stretch reflex depending on the spatial characteristics of the visual target that directly affects the requirements of the task (Mutha et al., 2008; Yang et al., 2011; Nashed et al., 2012). Meanwhile, in the current study, we manipulated the duration of the target presentation to increase its ambiguity without explicitly changing the task requirements. The results suggest that ambiguity in the target and uncertainty in the body state may be processed differently during modulation of the stretch reflex. Presumably, the latter may have a more crucial meaning for reflex control, considering that the key function of the stretch reflex is to maintain the body state in response to disturbances from the external world.

The control strategy underlying visually guided reaching movements has been investigated by examining how the removal of visual feedback affects motor behavior, including movement trajectory, endpoint error, movement time, and reaction time (Zelaznik et al., 1983; Khan et al., 2002; Hansen et al., 2006; Cheng et al., 2008). Although the previous studies have examined the effect of visual cursor and target presentation on movement precision, no attempt has been made to test the potential relationship with the modulation of the stretch-evoked motor response as investigated in the current study. It has been suggested that in a context in which participants are uncertain whether visual feedback will be available on the next trial (e.g., provided by trial-by-trial random switching of the presentation or removal of a visual cursor), participants tend to make a reaching movement without a visual cue on all trials as a “worst-case” preparation (Hansen et al., 2006; Elliott et al., 2017). The results of Experiment 2, however, showed a difference in endpoint variance between Cursor-on and Cursor-off trials or between Target-on and Target-off trials. This suggests that the participants still used the visual cue to control movement in Cursor-on and Target-on trials, even if the visual condition was randomly intermixed on a trial-by-trial basis. A possible reason for the use of the visual cue despite of uncertain trial conditions may be the randomness of the target location in Experiment 2, whereas in previous studies the target was displayed at single or multiple fixed locations. Possibly, this manipulation in the current study increased the need for a closed-loop control strategy using the visual cue (Elliott et al., 2017) to reduce the endpoint error on each trial, and may have resulted in the increase in endpoint error in trials where the visual cursor or target was not available. However, while the change in endpoint variability could be at least partially attributed to the above account, it does not explain the regulation of the stretch reflex observed in Cursor-off trials but not in Target-off trials. Thus, it is reasonable to assume that the modulation of the stretch reflex depends on visual uncertainty about the body state, but not on ambiguity about the target location.

Our experiments found a modulation in the response amplitude of the long-latency component but not in the short-latency component. These results are in accordance with the fact that context-dependent modulation of the stretch reflex is observed specifically in the long-latency components (Doemges and Rack, 1992; Kurtzer et al., 2008; Nashed et al., 2014). The long-latency stretch reflex has been shown to emerge from a transcortical pathway including primary motor and sensory cortices (Evarts and Tanji, 1976; Kurtzer, 2014; Scott et al., 2015) as well as the spinal network (Soteropoulos and Baker, 2020). Since the primary motor cortex is particularly involved in modulating the long-latency reflex in response to the task or body dynamics (Evarts and Tanji, 1976; Kimura et al., 2006; Pruszynski et al., 2011), it is natural to postulate that this area could be engaged in the neural mechanisms for generating the observed vision-dependent modulation. In addition, higher sensorimotor brain regions such as premotor or parietal cortex may also be responsible for the reflex tuning. A recent study has suggested that parietal area 5 is highly related to the state estimation and could be involved in regulation of gain quick feedback incorporated with primary motor cortex (Takei et al., 2021). Considering a view that the posterior parietal cortex, including area 5, is involved in state estimation that integrates visual cues (Buneo and Andersen, 2006; Medendorp and Heed, 2019), we could hypothesize that this region also contributes to the modulation of stretch reflex in response to visual feedback. To investigate these possibilities and to clarify the neurophysiological mechanisms, further studies are needed that integrates a computational account for the vision-dependent stretch reflex modulation.

The results of the two experiments support the hypothesis that the amplitude of the stretch reflex is regulated considering the uncertainty in the visual representation of the self-body state. Theoretical studies have proposed that the gain of feedback control is tuned in a context-dependent manner on the basis of estimates of the body state (Todorov and Jordan, 2002; Scott, 2004). Potentially, the state could be estimated for online feedback control by integrating multisensory information (Scott et al., 2015; Crevecoeur et al., 2016; Oostwoud Wijdenes and Medendorp, 2017). When visual feedback is unreliable, the body states estimated by integrating visual and proprioceptive information also become less reliable, even though the optimal sensory integration process maintains as much reliability as possible (Ernst and Banks, 2002; van Beers et al., 2002). Possibly, the observed regulation of the stretch reflex may be the result of a brain function to reduce the risk of generating an incorrect motor response based on an erroneous body state when the estimated value is not reliable. In addition, another interpretation is that a decrease in the stretch reflex protects the body from damage caused by unintended contact with the external world. High uncertainty in estimating the body state means an increase in the risk of a collision between the body and an external object. In this situation, lower limb impedance should be preferable in case of unintended contact. Given that the stretch reflex is known to contribute to the modulation of limb impedance (Krutky et al., 2010), reducing its gain could functionally work when visual uncertainty is high and state estimation is unreliable.

In summary, the present study showed a modulation of the stretch reflex by manipulating visual information regarding the body state. The results suggest the involvement of visually induced uncertainty in hand states in regulating the stretch reflex. The findings provide insight into brain mechanisms for monitoring states of the body and environment underlying functional tuning of quick feedback control.

## Data availability statement

The raw data supporting the conclusions of this article will be made available by the authors, without undue reservation.

## Ethics statement

The studies involving humans were approved by the NTT Communication Science Laboratories Ethics Committee. The studies were conducted in accordance with the local legislation and institutional requirements. The participants provided their written informed consent to participate in this study.

## Author contributions

SI: Conceptualization, Data curation, Formal Analysis, Writing – original draft. HG: Conceptualization, Supervision, Writing – review and editing, Funding acquisition.
